# The Latest Discovery

**Published:** 1897-04

**Authors:** 


ARTICLE IV.

### THE LATEST DISCOVERY.

The discovery of acetyline gas appears to have struck capitalists all at once, and in many different parts of the country. It is to be introduced simultaneously at New York, Philadelphia and Chicago, and we shall doubtless soon hear of it in this city. Its advantages may be summarized in a single sentence—it is an illuminant which, for the same money, produces a light forty times as brilliant as ordinary city gas, and far more brilliant than the ordinary electric light. It creates high noon at midnight.

Acetyline is formed from its elements, carbon and hydrogen, when the electric arc is passed between carbon points in an atmosphere of hydrogen. It has been known for many years, but the experiments with it have been checked by its poisonous effects on life and the known tendency of many of its compounds to explode. Chemists have now succeeded in obviating these objections to its use, and it is probably destined to extensive employment in various ways. A plant is being erected at Niagara Falls, at which, by the use of water-power, it is expected that twenty-five tons of the substance from which acetyline gas is made can be produced daily at a cast of less than $20 per ton. It is figured that it will presently be turned out for considerably less than a cent a pound, at which price the gas would come into general use as an illuminant.

This is only one of the uses in which the new gas can be put. Acetyline will produce whole systems of dyes, medicines, essences, perfumes, poisons, explosives, to say nothing of alcohol, so that besides helping the human race it will confer upon it a more questionable benefit in supplying it with cheap whiskey.

If acetyline gas is passed through an iron tube heated to dull redness, it turns into benzine, which is the base of thousands of organic substances. If benzine vapor be passed into strong nitric acid, it is transformed into nitrobenzine, and by treating this with hydrochloric acid, aniline is produced and with it the immense series of dyes of which it is the base. Another chemical process will convert acetyline into carbolic acid and picric acid, which has superseded nitric acid in the manufacture of high explosives. Yet another process, in which aniline is boiled with acetic acid, leads to the production of antifebrin, which is now so largely used in lowering the action of the heart and checking fever and headache. Yet another process treats acetyline with nascent hydrogen; the product is etheline, which, when mixed with sulphuric acid and water, turns into alcohol. In fact, it is difficult to enumerate all the uses to which the various compounds of acetyline may be put.

It is a privilege to live in an age when science is constantly making life easier and more convenient than it was. And it is instructive to observe that while each new discovery displaces some established industry and throws men and industry out of employment, new fields of usefulness are always found to fill the vacuum. When railroads were planned, it was said that there would be no more call for horses; yet they now command more money than ever. When the aniline dyes superseded vegetables indigo and animal cochineal, it was said that the regions which produced those coloring pigments would be deserted, but they are more prosperous than ever. Acetyline gas will probably give the death blow to coal gas, but at the same time the uses for coal are expanding with the extension of manufacturing which acetyline will inspire, and thus the loss which is occasioned on one side will be more than offset by the gain realized on another.—*Pacific Record of Medicine and Surgery.*

